# *Klebsiella pneumoniae* infection induces an S100A8/A9-mediated autocrine loop in human airway epithelium to amplify inflammation

**DOI:** 10.3389/fmicb.2026.1768140

**Published:** 2026-03-03

**Authors:** Jie Zheng, Xiuchan Feng, Qianrui Zeng, Shutong Chen, Caihong Yan, Zhijia Huang

**Affiliations:** 1 Department of Anesthesiology, The Second Affiliated Hospital, Hengyang Medical College, University of South China, Hengyang, China; 2 Department of Critical Care Medicine, The Second Affiliated Hospital, Hengyang Medical College, University of South China, Hengyang, China; 3 Hunan Provincial Key Laboratory for Special Pathogens Prevention and Control, Institute of Pathogenic Biology, Hengyang Medical College, University of South China, Hengyang, China

**Keywords:** bronchial epithelial cells, *Klebsiella pneumoniae*, S100A8, S100A9, toll-like receptor 4 (TLR4)

## Abstract

*Klebsiella pneumoniae* pneumonia is marked by an excessive inflammatory response that drives lung injury. The initial stages of infection and the mechanisms driving hyper-inflammation at the airway epithelial barrier are not fully defined. The alarmins S100A8/A9 are potent inflammatory mediators, and studies have indicated their importance in the host response to *K. pneumoniae*. Here, we show that infection with live *K. pneumoniae* directly induces the transcription and secretion of the S100A8/A9 heterodimer in a primary human bronchial epithelial (HBE) cell model. Furthermore, the infection sensitizes epithelial cells by upregulating the expression of Toll-like receptor 4, a key receptor that mediates S100A8/A9 signaling. We also established that HBE cells are highly responsive to extracellular S100A8/A9, which stimulates pro-inflammatory cytokine production. Critically, silencing of endogenous S100A9 expression in HBE cells significantly attenuated NF-κB activation and the overall cytokine response to bacterial infection, providing direct evidence for an epithelium-intrinsic autocrine amplification loop. Functionally, disruption of this loop markedly reduced the ability of epithelial cell supernatants to induce neutrophil chemotaxis. These findings reveal a novel autocrine mechanism that positions the airway epithelium not just as a responder, but as an active initiator and amplifier of local inflammation during early *K. pneumoniae* infection.

## 1 Introduction

*Klebsiella pneumoniae* has emerged as a formidable pathogen in both hospital- and community-acquired settings, responsible for severe pneumonia, sepsis, and a spectrum of invasive diseases (Zhou et al., 2022; Grosjean et al., 2024). A hallmark of severe *K. pneumoniae* pneumonia is a rapid and excessive inflammatory response within the lungs (Dulger et al., 2025). While inflammation is a critical host defense mechanism for bacterial clearance, its dysregulation often leads to extensive tissue damage, acute lung injury (ALI), and acute respiratory distress syndrome (ARDS) (Huang et al., 2023; Li et al., 2024), suggesting that the host immune response is a major driver of disease severity.

The airway epithelium constitutes the primary interface between the host and inhaled pathogens, functioning not merely as a physical barrier but as a crucial sentinel of the innate immune system (He et al., 2024). Upon encountering microbial threats, epithelial cells initiate protective responses, including the secretion of cytokines and chemokines to recruit professional immune cells (Cortes et al., 2002; Barclay et al., 2023). Understanding the earliest molecular events at this epithelial barrier is therefore pivotal to deciphering the mechanisms that determine whether an immune response remains protective or escalates into destructive hyper-inflammation.

S100A8 and S100A9 are calcium-binding proteins that form a heterodimer (S100A8/A9, or calprotectin), which functions as a potent endogenous danger-associated molecular pattern (DAMP) or alarmin (Wang et al., 2018; Tan et al., 2025). Traditionally, S100A8/A9 is known to be abundantly expressed and released by myeloid cells upon activation at sites of infection (Foell et al., 2007; Bai et al., 2021). Extracellular S100A8/A9 dramatically amplifies inflammation by engaging pattern recognition receptors, primarily Toll-like receptor 4 (TLR4) and the receptor for advanced glycation end products (RAGE), thereby stimulating further cytokine production and leukocyte recruitment (Henke et al., 2006; Ma et al., 2017). While the function of S100A8/A9 originating from myeloid cells in various bacterial infections is well-established, its function during *K. pneumoniae* infection appears complex, with studies in murine models pointing towards a protective, antimicrobial role systemically (Revenstorff et al., 2022). Although S100A8/A9 expression within the respiratory epithelium has been documented in chronic conditions such as chronic obstructive pulmonary disease (COPD) and asthma, and has been shown to be induced by bacterial components like lipopolysaccharide (LPS) (Henke et al., 2006), most of these studies have focused on its role as a marker of chronic inflammation or its capacity to modulate professional immune cells like macrophages (Skronska-Wasek et al., 2022). However, the specific temporal dynamics and the potential for a self-amplifying, epithelium-intrinsic circuit triggered specifically during the acute phase of *K. pneumoniae* infection remain largely undefined. Specifically, whether the alarmin and its signaling receptors are concurrently upregulated to orchestrate a localized inflammatory burst has not been established.

This led us to investigate the specific contribution of epithelium-derived S100A8/A9 to the localized inflammatory output during the initial stages of *K. pneumoniae* infection. We hypothesize that *K. pneumoniae* establishes a priming and triggering environment by directly inducing HBE cells to synthesize S100A8/A9 while concurrently upregulating its cognate receptor, TLR4. We further propose that this epithelial-intrinsic loop then acts in an autocrine manner to drive the excessive cytokine production and subsequent neutrophil recruitment that characterize the acute immunopathology of severe pneumonia.

In this study, we utilized primary HBE cells to model the initial host-pathogen interaction. We demonstrate that live *K. pneumoniae* infection robustly induces the expression and secretion of S100A8/A9 from these cells. We show that this epithelial-derived S100A8/A9 is functionally essential for the full inflammatory cytokine response to infection. Finally, we delineate the underlying signaling mechanism, revealing that the S100A8/A9-driven amplification loop is mediated through TLR4 and the canonical NF-κB pathway. Our findings identify a novel, epithelium-intrinsic mechanism that initiates and perpetuates inflammation in the lung, positioning epithelial-derived S100A8/A9 as a key driver of early immunopathology in *K. pneumoniae* pneumonia.

## 2 Materials and methods

### 2.1 Materials

Endotoxin-free S100A8/A9 heterodimer (Calprotectin) was purchased from R&D Systems (Cat# 2220-S8-050). The following function-blocking monoclonal antibodies or western blotting antibodies were used: anti-TLR4 (InvivoGen Cat# mabg-htlr4-2) and anti-RAGE (R&D Systems, Cat # 1145-RG). Anti-total IκBα (Cat# 4814) and *β*-actin (Cat# 4970) were purchased from Cell Signaling Technology. HRP-conjugated secondary antibodies were from Cell Abcam.

### 2.2 Cell culture

Primary HBE cells were obtained from Lonza (Cat# CC-2540) and cultured in BEGM medium (Lonza, Cat# CC-3170) as described previously (Qin et al., 2022). For experiments, HBE cells were cultured onto collagen-coated plates (Corning) and grown to 80–90% confluence. Twenty-four hours prior to bacterial infection or stimulation, the culture medium was replaced with antibiotic-free BEGM basal medium supplemented with only the BEGM SingleQuots™ Triiodothyronine, Transferrin, and Insulin (T/T/I) supplements to minimize confounding effects from serum or other growth factors.

### 2.3 Bacterial strains and culture conditions

*Klebsiella pneumoniae* serotype K2 (ATCC 43816) was used as the wild-type (WT) strain. An isogenic acapsular mutant (Δ*cps*) of a K2 strain, a gift of Dr. Lisong Chen (University of South China), was used as a control. Bacteria were grown from frozen glycerol stocks in LB broth at 37 °C for 12 h with shaking (220 rpm). For infection experiments, the overnight culture was subcultured at a 1:100 ratio in fresh LB broth and grown to mid-logarithmic phase. Cells were centrifugated and washed with PBS, and resuspended in antibiotic-free BEBM basal medium. Concentration was determined from OD₆₀₀ (1.0 ≈ 1 × 10^9^ CFU/mL) and confirmed by plating.

### 2.4 *In vitro* infection of *Klebsiella pneumoniae* and S100A8/A9 stimulation

Prior to infection, HBE cell monolayers at 80–90% confluence were washed with PBS. Bacteria were inoculated at various Multiplicity of Infection (MOI) and infection was synchronized via low-speed centrifugation before cultured at 37 °C in 5% CO₂. For S100A8/A9 stimulating experiment, cells were incubated with endotoxin-free recombinant human S100A8/A9 protein (R&D Systems) at the indicated concentrations for specified durations. Culture supernatants were harvested for cytokine assays; or cells were lysed for PCR or western blotting.

### 2.5 Quantitative PCR

Following RNA extraction from HBE cells using the RNeasy Mini Kit (QIAGEN) with on-column DNase I digestion, total RNA was quantified and 1 μg was reverse-transcribed into cDNA (Applied Biosystems, Cat#4368814). Gene expression was quantified by real-time PCR on a StepOnePlus system with SYBR Green Master Mix (Applied Biosystems, Cat#A25742). Data were analyzed using the 2^−ΔΔCt^ method (Luo et al., 2021). For genes showing a decrease in expression (where 2^−ΔΔCt^<1), the results are reported as fold reduction by calculating the negative inverse of the fold change (−1/2^−ΔΔCt^), according to the standard protocol by Schmittgen and Livak (2008). All primer sequences are provided in Table 1 (Sangon Biotech) (Vogl et al., 2018).

**Table 1:** Primers used for quantitative PCR analysis.

Gene	Forward primer (5’→3’)	Reverse primer (5’→3’)
*S100A8*	ATGCCGTCTACAGGGATGACCT	AGAATGAGGAACTCCTGGAAGTTA
*S100A9*	GCACCCAGACACCCTGAACCA	TGTGTCCAGGTCCTCCATGATG
*TLR4*	CCAGAACCAAACGATGGACT	CCTGTCCCTGAACCCTATGA
*IL-8*	GAGAGTGATTGAGAGTGGACCAC	CACAACCCTCTGCACCCAGTTT
*IL-6*	AGAGGCACTGGCAGAAAACAAC	AGGCAAGTCTCCTCATTAATCC
*GAPDH*	CAAATTCCATGGCACCGTCA	TCGCCCCACTTGATTTTGG

### 2.6 S100A8/A9 and cytokines detection

Cell culture supernatants were collected at specified time points. The concentrations of secreted S100A8/A9 heterodimer, IL-8, and IL-6 were quantified by commercial Quantikine ELISA kits (R&D Systems, Cat# DS8900 for S100A8/A9, D8000C for IL-8, and D6050B for IL-6). Following the kit instructions, absorbance was measured (F50, TECAN).

### 2.7 siRNA-mediated gene silencing

To overcome the transfection challenge in primary epithelial cells, HBE cells were subjected to siRNA-mediated S100A9 knockdown via the Amaxa™ 4D-Nucleofector™ System (Lonza). For each electroporation, 2 × 10^6^ cells in 100 μL P3 Primary Cell Solution were combined with 200 pmol of S100A9-targeting or non-targeting control siRNA (Horizon Discovery). After applying the manufacturer’s recommended pulse program for airway cells, transfected cells were replated in BEGM medium and allowed to recover for 48 ~ 72 h. Successful knockdown was validated at both the transcriptional (qRT-PCR) and secretory protein (ELISA) levels.

### 2.8 Receptor blocking assays

To block specific receptors, HBE cells were separately pre-treated for 1 h with either an anti-human TLR4 (10 μg/mL) or an anti-human RAGE (100 μg/mL) neutralizing antibody, or their respective isotype controls prior to stimulation with recombinant S100A8/A9. Following stimulation, supernatants were collected for cytokine measurement by ELISA.

### 2.9 Western blot analysis

Cells were lysed in RIPA buffer containing protease and phosphatase inhibitors. Following protein quantification (BCA assay), samples (20–30 μg) were subjected to SDS-PAGE and transferred to PVDF membranes. For immunodetection, membranes were blocked in 5% milk and then incubated with specific primary antibodies overnight at 4 °C. After washing, HRP-conjugated secondary antibodies were applied, and proteins were visualized by chemiluminescence (SuperSignal™ West Pico PLUS) on a ChemiDoc™ imager.

### 2.10 Immunofluorescence

Following transfection and infection on coverslips, HBE cells were fixed, permeabilized, and blocked. NF-κB p65 was detected using a specific primary antibody and an Alexa Fluor 488-conjugated secondary antibody, with DAPI to identify nuclei. Imaging was performed on a Nikon Ts2R microscope.

### 2.11 Neutrophil chemotaxis assay

Primary human neutrophils were isolated from healthy donors via Ficoll-Paque density gradient centrifugation. Chemotaxis assays were performed using Transwell inserts with a 3 μm pore size. Conditioned media (CM) were harvested from HBE cells following 24 h of infection or specific treatments. CM samples or fMLP (100 nM, as a positive control) were loaded into the lower chambers, and neutrophils (5 × 10^5^ cells) were introduced into the upper chambers and allowed to migrate for 90 min at 37 °C. Transmigrated cells were collected and quantified by flow cytometry (BD FACSCalibur) using CountBright™ Absolute Counting Beads (Thermo Fisher Scientific) as an internal standard to determine absolute cell counts relative to the beads within a defined gate.

### 2.12 Ethics statement

The study was conducted in accordance with the Declaration of Helsinki. The study protocol involving human participants was reviewed and approved by the Ethics Committee of the University of South China (Approval No. 2023–007). All healthy donors provided informed written consent prior to blood collection for the isolation of primary neutrophils.

### 2.13 Statistical analysis

Data represent at least three biologically independent experiments. Values are shown as mean ± SEM. Statistical analyses were performed using GraphPad Prism 9 Software. Comparisons between two groups were made using an unpaired, two-tailed Student’s *t*-test. Comparisons among three or more groups were performed using one-way or two-way analysis of variance (ANOVA) followed by Tukey’s or Dunnett’s *post-hoc* test for multiple comparisons. *p* < 0.05 was considered statistically significant.

## 3 Results

### 3.1 *Klebsiella pneumoniae* infection directly induces *S100A8/A9* transcription and secretion in HBE cells

**Figure 1:**
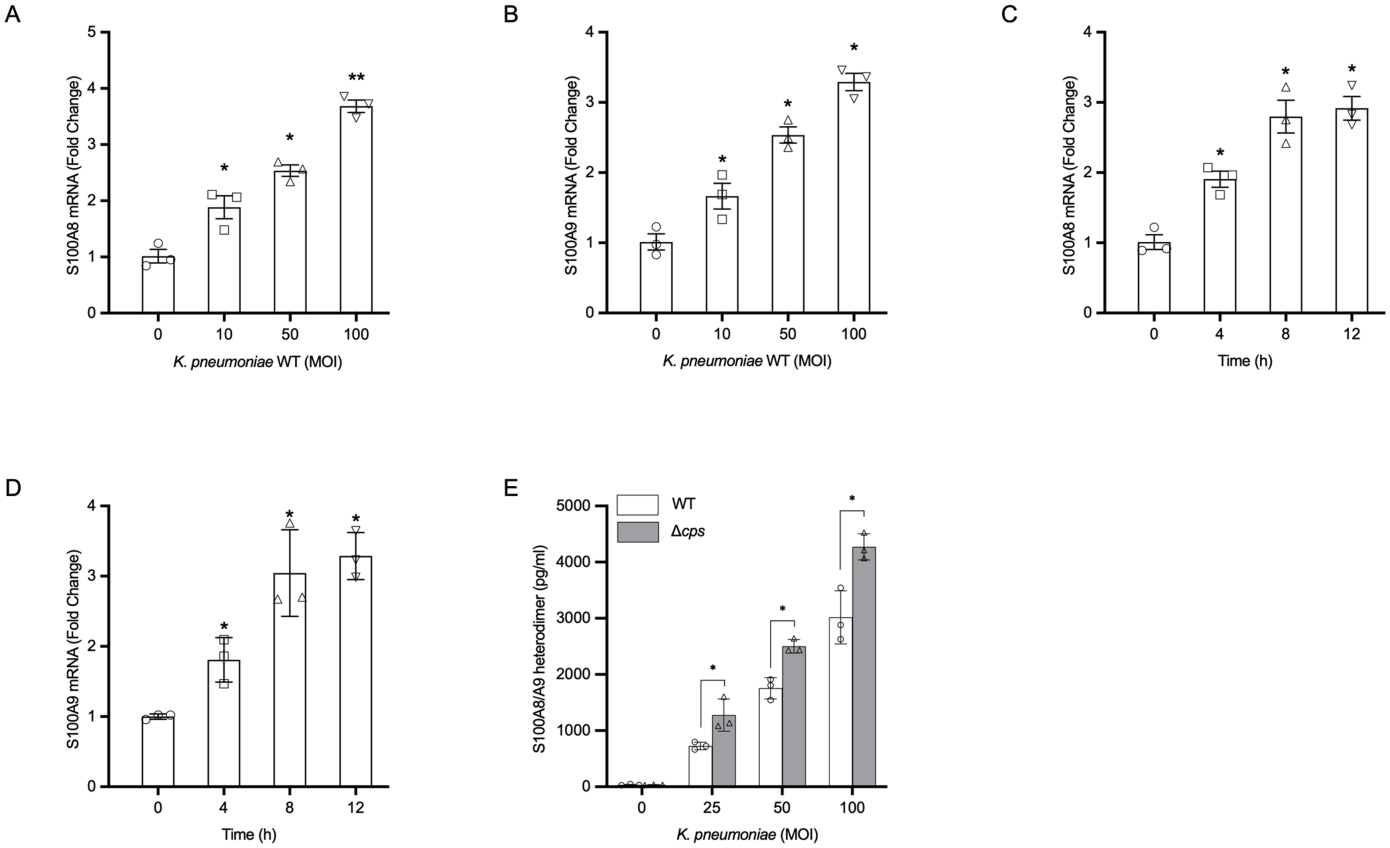
*Klebsiella pneumoniae* infection directly induces S100A8/A9 expression and secretion in HBE cells. Primary HBE cells were infected with WT *K. pneumoniae* or an isogenic acapsular mutant (Δ*cps*). **(A,B)** Expression of *S100A8* and *S100A9* was determined by qRT-PCR at 8 h post-infection with various MOIs of *K. pneumoniae*, or 100 MOI *K. pneumoniae* for 4–12 h **(C,D)**. Data are normalized to the housekeeping gene *GAPDH* and expressed as fold change relative to uninfected control cells. **(E)** Secretion of S100A8/A9 heterodimer induced by WT *K. pneumoniae* and the isogenic Δ*cps* mutant at indicated MOIs after 24 h. All data are presented as mean ± SEM of three independent experiments. *p < 0.05, **p < 0.01 vs. uninfected cells **(A–D)**. Statistical differences between the WT and Δ*cps* groups were determined by Two-way ANOVA followed by Tukey’s *post-hoc* test. *p < 0.05 compared between strains at the same MOI **(E)**.

To investigate whether the airway epithelium is a direct source of the alarmin S100A8/A9 during bacterial infection, HBE cells were infected with wild-type *K. pneumoniae*. Quantitative RT-PCR showed that *K. pneumoniae* infection for 8 h induced an MOI-dependent increase in the mRNA of *S100A8* and *S100A9* (Figures 1A,B). Furthermore, the transcription of *S100A8* and *S100A9* exhibited a time-dependent increase following infection with *K. pneumoniae* at an MOI of 100 (Figures 1C,D). This transcriptional upregulation was followed by a marked increase in the secretion of the S100A8/A9 heterodimer into the culture supernatant, as quantified by ELISA (Figure 1E). A direct statistical comparison between the bacterial strains revealed that the isogenic acapsular mutant (Δ*cps*) elicited significantly higher levels of S100A8/A9 protein secretion compared to the WT strain at equivalent MOI (Figure 1E). These data provide concrete evidence that the bacterial capsule functions as a physical shield that masks the bacterial ligands responsible for triggering the alarmin response in HBE cells.

### 3.2 Extracellular S100A8/A9 functions as a potent pro-inflammatory stimulus for HBE cells

**Figure 2:**
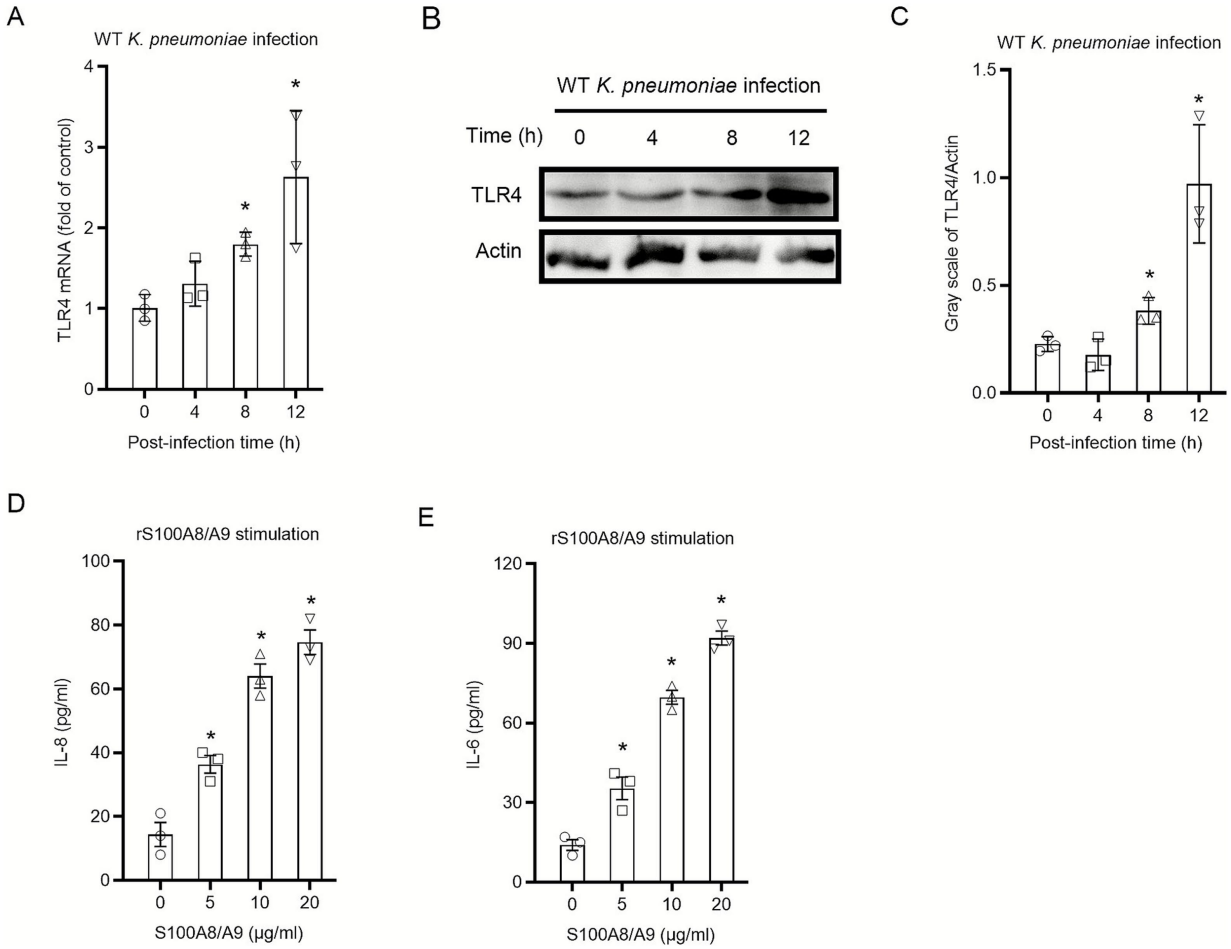
Extracellular S100A8/A9 functions as a potent pro-inflammatory stimulus for primary HBE cells. **(A)** Primary HBE cells were infected with WT *K. pneumoniae* (MOI 100) for the indicated time points. Relative mRNA expression of *TLR4* was determined by qRT-PCR, with data calibrated to GAPDH and shown as fold induction over the baseline (0 h). **(B)** Representative western blot showing total TLR4 protein levels. *β*-Actin served as the housekeeping protein. **(C)** Quantification of TLR4 protein expression from three independent replicates, with values standardized to β-Actin. **(D,E)** HBE cells were treated for 24 h with the specified doses of endotoxin-free recombinant human S100A8/A9 (rS100A8/A9). The release of **(D)** IL-8 and **(E)** IL-6 was detected by ELISA. Data are presented as mean ± SEM of three independent experiments. *p < 0.05 versus the untreated control **(A,C,D,E)**.

To explore whether bacterial infection could alter the sensitivity of epithelial cells to endogenous alarmins, the expression of the S100A8/A9 receptor TLR4 was examined post-infection. Following infection of HBE cells with WT *K. pneumoniae*, qRT-PCR analysis demonstrated a time-dependent upregulation of *TLR4* mRNA, which peaked at 12 h, a trend that was also observed for total TLR4 protein by Western blot (Figures 2A–C). We next sought to determine if HBE cells, in addition to producing S100A8/A9, could also respond to it. Uninfected HBE cells were treated with recombinant human S100A8/A9. This stimulation resulted in a robust secretion of the IL-8 and IL-6 (Figures 2D,E). These data suggest that *K. pneumoniae* infection not only induces the production of S100A8/A9 but also enhances the expression of its cognate receptor, potentially sensitizing the epithelium to subsequent S100A8/A9 signaling.

### 3.3 Epithelial-derived S100A8/A9 is functionally required for the full inflammatory response to *Klebsiella pneumoniae* infection

**Figure 3:**
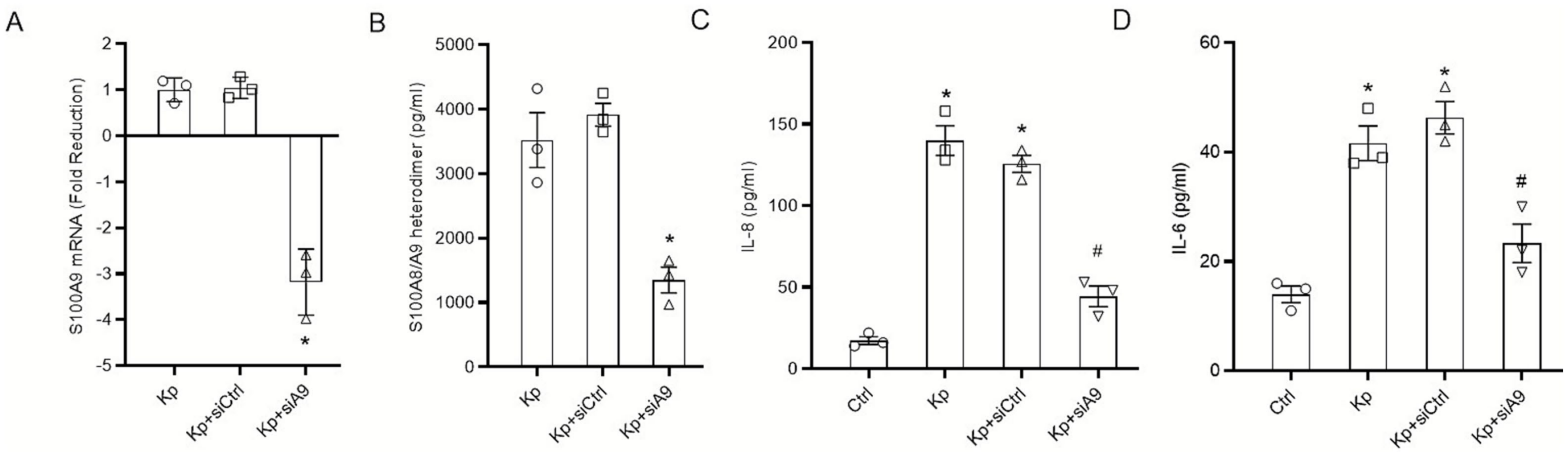
Epithelial-derived S100A8/A9 is functionally required for the full inflammatory response to *K. pneumoniae* infection. Primary HBE cells were transfected with control siRNA (siCtrl) or *S100A9* siRNA (siA9). **(A)** Knockdown efficiency was confirmed 48 h post-transfection by qRT-PCR analysis of *S100A9* mRNA levels following a 12-h infection with WT *K. pneumoniae* (MOI 100), negative values represent fold reduction calculated as the negative inverse of 2^−ΔΔCt^ following the Schmittgen and Livak protocol. **(B)** Functional knockdown was confirmed by measuring S100A8/A9 protein secretion by ELISA in supernatants from infected cells at 24 h. **(C,D)** Control and *S100A9*-deficient cells were infected with WT *K. pneumoniae* (MOI 100) for 24 h, and the secretion of cytokines were quantified by ELISA. Data are presented as mean ± SEM of three independent experiments. ^*^p < 0.05 vs. Kp + siCtrl **(A,B)** or uninfected cells **(C,D)**, ^#^*p* < 0.05 vs. Kp + siCtrl **(C,D)**.

To establish the functional importance of epithelial-derived S100A8/A9, siRNA was used to specifically silence *S100A9* expression in HBE cells, which prevents the formation of the stable heterodimer. Successful knockdown was validated by qRT-PCR, showing a 3.18-fold reduction in S100A9 mRNA levels compared to control siRNA-transfected cells (Figure 3A), which was consistent with the decreased S100A8/A9 protein secretion (Figure 3B). To ensure that the attenuated inflammation was not due to altered bacterial load, we confirmed that S100A9 silencing did not affect the initial adherence or internalization of *K. pneumoniae* (Supplementary Figure S1A). Following infection with WT *K. pneumoniae*, S100A9-deficient cells exhibited a significantly attenuated inflammatory response, with a 65% reduction in IL-8 and a 50% reduction in IL-6 secretion compared to control cells (Figures 3C,D). Critically, this inhibitory effect was specific to bacterial challenge, as S100A9 deficiency did not impair the cytokine response to a non-bacterial stimulus, TNF-α (Supplementary Figures S1B,C). These findings collectively demonstrate that the epithelial S100A8/A9-TLR4 axis is a mandatory component of the early inflammatory cascade during infection.

### 3.4 TLR4 is the mandatory receptor for both exogenous and infection-induced S100A8/A9 signaling

**Figure 4:**
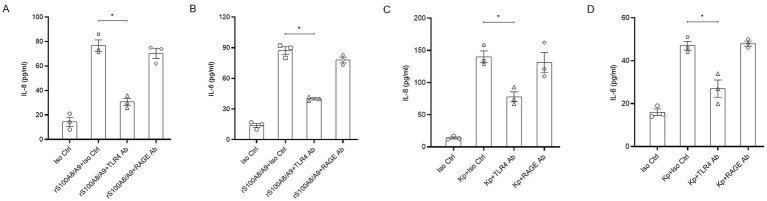
TLR4 is the essential receptor mediating S100A8/A9-induced inflammation in HBE cells during *K. pneumoniae* infection. **(A,B)** HBE cells were pre-incubated with neutralizing antibodies against TLR4 (10 μg/mL), RAGE (100 μg/mL), or an isotype control (Iso Ctrl) for 1 h, followed by stimulation with recombinant human S100A8/A9 (20 μg/mL) for 24 h. Levels of **(A)** IL-8 and **(B)** IL-6 in supernatants were determined by ELISA. **(C,D)** HBE cells were pre-treated with the indicated antibodies for 1 h and subsequently infected with WT *K. pneumoniae* (MOI 100) for 24 h. Secretion of **(C)** IL-8 and **(D)** IL-6 was quantified by ELISA. All results are expressed as mean ± SEM of three independent experiments. *p < 0.05 compared with the indicated groups.

Previous studies have established that the S100A8/A9 heterodimer can signal through either TLR4 or RAGE (Kang et al., 2015; Ma et al., 2017). To identify the specific receptor facilitating the autocrine loop in HBE cells, we performed targeted blocking assays. Initially, we tested the effect of exogenous rS100A8/A9 stimulation. Pre-incubation with a neutralizing anti-TLR4 antibody significantly abrogated the secretion of IL-8 and IL-6 induced by rS100A8/A9 (Figures 4A,B). In contrast, an anti-RAGE antibody had no significant inhibitory effect (Figures 4C,D). Critically, to validate this axis during an active infection, we performed receptor blockade during *K. pneumoniae* challenge. As shown in Figures 4C,D, the robust cytokine burst triggered by *K. pneumoniae* was significantly attenuated by the anti-TLR4 antibody, whereas the anti-RAGE antibody provided no protection. These results, confirm that TLR4 is the principal and mandatory signaling conduit for both the alarmin protein itself and the endogenous alarmin loop initiated by bacterial infection.

### 3.5 The S100A8/A9 autocrine loop activates the canonical NF-κB signaling pathway during *Klebsiella pneumoniae* infection

**Figure 5:**
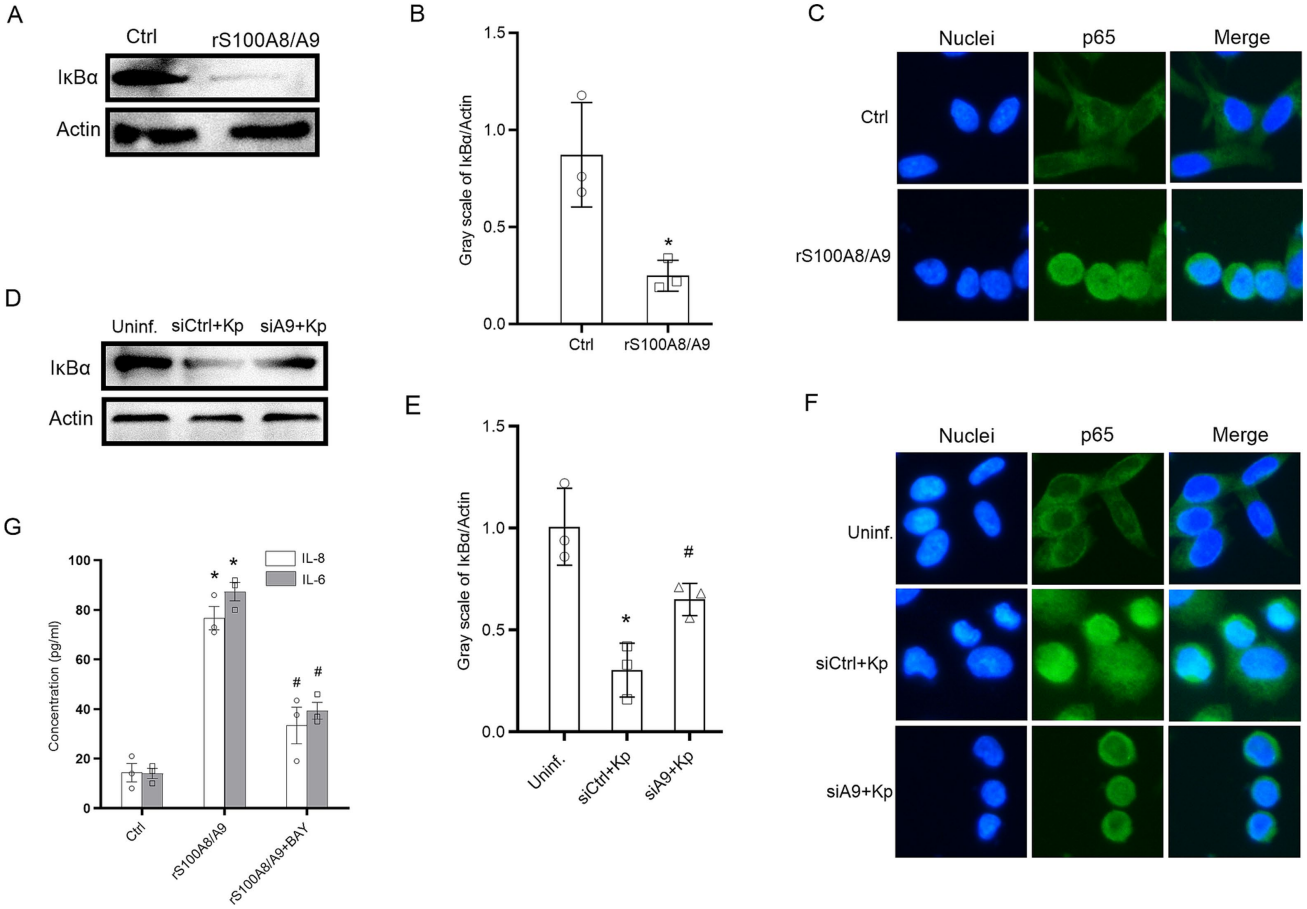
The S100A8/A9 autocrine loop drives NF-κB activation and subsequent cytokine release in HBE cells. **(A)** Representative immunoblots showing degradation of IκBα in primary HBE cells stimulated with rS100A8/A9 (20 μg/mL) for 60 min. β-Actin served as a loading control. **(B)** Densitometric quantification of IκBα protein levels from **(A)**. **(C)** Representative immunofluorescence images showing subcellular localization of the p65 subunit (green) at 60 min post-stimulation with rS100A8/A9. Nuclei were counterstained with DAPI (blue). **(D)** HBE cells were transfected with control siRNA (siCtrl) or siRNA targeting S100A9 (siS100A9) and subsequently infected with wild-type *K. pneumoniae* (MOI 100). Representative immunoblots of IκBα levels are shown at 60 min post-infection. **(E)** Densitometric quantification of IκBα degradation from **(D)**. **(F)** Representative immunofluorescence images showing p65 localization (green) in siCtrl- or siS100A9-transfected HBE cells at 60 min post-*K. pneumoniae* infection. Nuclei were stained with DAPI (blue). **(G)** Secretion of IL-8 and IL-6 by HBE cells. Cells were pre-treated with or without the NF-κB inhibitor BAY 11–7,082 (10 μM) for 60 min, followed by stimulation with rS100A8/A9 (20 μg/mL) for 24 h. Cytokine levels in supernatants were measured by ELISA. All graphs display mean ± SEM, *p < 0.05 compared with the untreated control **(B,E,G)**; ^#^*p* < 0.05 compared with siCtrl + Kp or the S100A8/A9-stimulated group without inhibitor **(E,G)**.

To delineate the molecular mechanism of the alarmin-mediated amplification loop, we utilized two complementary approaches. First, to determine if S100A8/A9 is a direct activator of the signaling cascade, uninfected HBE cells were stimulated with rS100A8/A9. This exogenous challenge triggered the degradation of IκB and the nuclear translocation of p65 (Figures 5A–C), confirming the potent signaling potential of alarmin. Second, to assess whether the endogenous alarmin loop is required for pathway activation during the host-pathogen interaction, we monitored NF-κB dynamics during *K. pneumoniae* infection in S100A9-deficient cells. As expected, the robust signaling response induced by *K. pneumoniae* was significantly attenuated when the endogenous alarmin system was silenced (Figures 5D,E). Consistent with these biochemical findings, immunofluorescence analysis revealed that the nuclear translocation of p65 induced by *K. pneumoniae* was markedly reduced in S100A9-deficient cells compared to control cells (Figure 5F). These results collectively demonstrate that the S100A8/A9 autocrine loop is the principal driver of strong NF-κB activation in epithelial cells following *K. pneumoniae* infection. Finally, to establish a direct functional link between this signaling axis and cytokine release, we utilized the pharmacological NF-κB inhibitor BAY 11–7,082. As shown in Figure 5G, pre-treatment with BAY 11–7,082 significantly abrogated the secretion of IL-8 and IL-6 induced by rS100A8/A9 stimulation. These data provide robust functional confirmation that the canonical NF-κB pathway is the mandatory signaling conduit for this amplification loop.

### 3.6 The epithelial S100A8/A9 autocrine loop is a critical driver of neutrophil chemotaxis in response to *Klebsiella pneumoniae* infection

**Figure 6:**
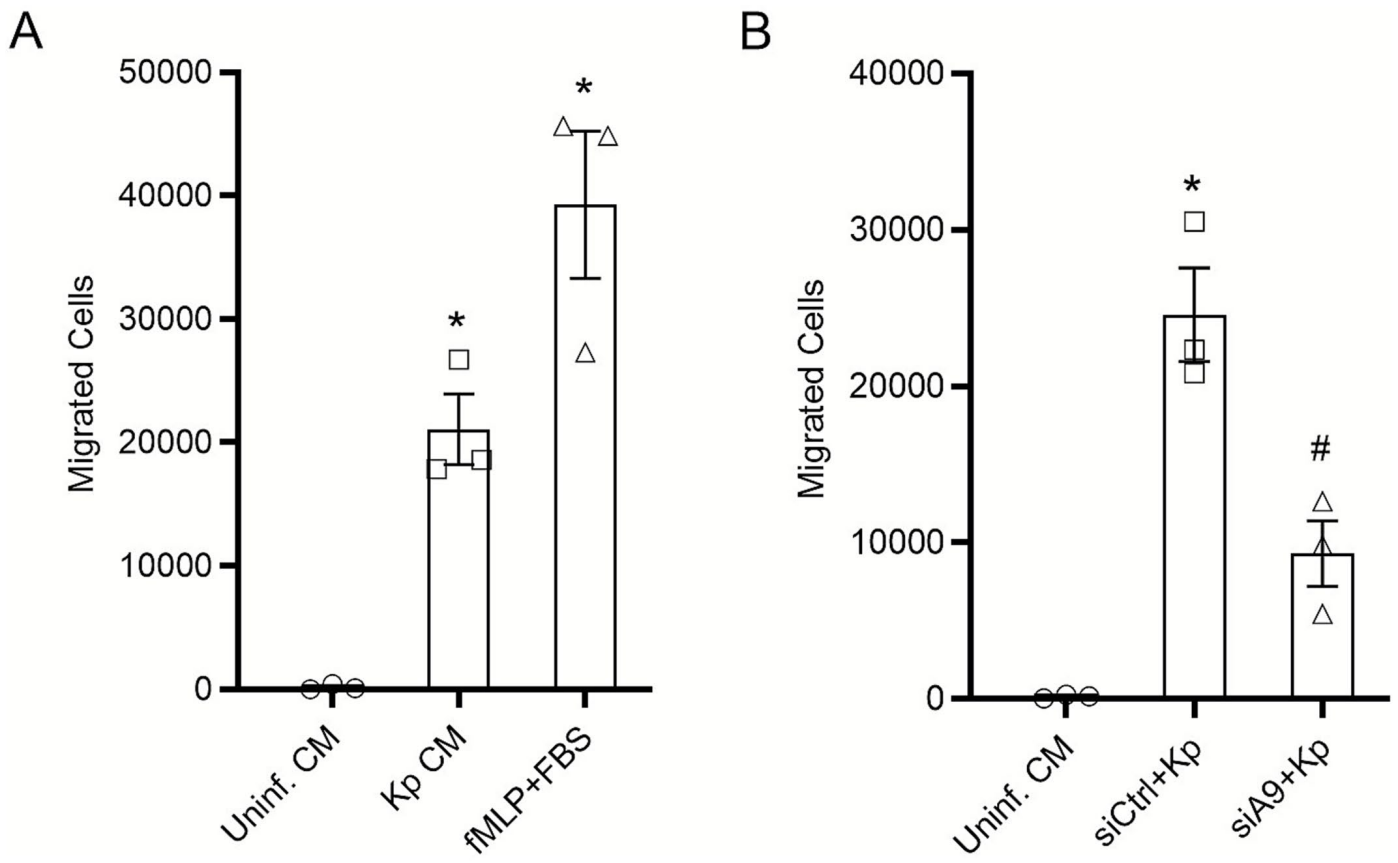
The epithelial S100A8/A9 autocrine loop is a critical driver of neutrophil chemotaxis in response to *K. pneumoniae* infection. Neutrophil chemotaxis was assessed using a Transwell assay. **(A)** Comparison of the chemotactic activity of supernatants from uninfected HBE cells (conditioned medium, CM), HBE cells infected with WT *K. pneumoniae* (MOI 100), and fMLP (positive control) on primary human neutrophils. **(B)** Comparison of the chemotactic activity of supernatants from infected control (siCtrl) HBE cells versus infected S100A9-deficient (siS100A9) HBE cells. Flow cytometry was used to determine the absolute number of migrated neutrophils. Data are shown as mean ± SEM from three separate experiments. *p < 0.05 vs. uninfected CM, ^#^*p* < 0.05 vs. Kp + siCtrl.

To determine the functional consequence of the epithelium-intrinsic S100A8/A9 amplification loop, we investigated whether supernatants from infected HBE cells could recruit neutrophils. A Transwell migration assay was performed using freshly isolated primary human neutrophils, with migrated cells quantified by flow cytometry. As shown in Figure 6A, conditioned supernatants from HBE cells infected with WT *K. pneumoniae* induced robust and significant neutrophil migration, which was substantially greater than that induced by supernatants from uninfected cells. Crucially, to establish the specific role of the epithelial S100A8/A9 loop in this process, we tested supernatants from S100A9-deficient HBE cells. The chemotactic potential of supernatants from infected, S100A9-deficient cells was significantly diminished, with the number of migrated neutrophils reduced by approximately 60% versus controls (Figure 6B). Collectively, these findings position the S100A8/A9-TLR4 autocrine signaling axis within the epithelium as a key mechanism for orchestrating neutrophil influx to infection sites.

## 4 Discussion

The data we report establish a previously unrecognized role for the airway epithelium as an active initiator and amplifier of inflammation during the early stages of *Klebsiella pneumoniae* infection. A key finding of this study is the identification of an epithelium-intrinsic S100A8/A9-driven autocrine loop. This study demonstrates not only that primary human bronchial epithelial cells are a source of S100A8/A9 upon bacterial challenge, but also that this endogenously produced alarmin is functionally required for the full NF-κB-driven inflammatory response and consequent cytokine production. This positions the epithelium as a key player in setting the inflammatory tone at the site of infection, independent of the immediate infiltration of professional immune cells.

A significant finding of our study is the demonstration that *K. pneumoniae* infection itself sensitizes epithelial cells for this amplified response by upregulating the expression of TLR4. Specifically, we observed that both *TLR4* mRNA and protein levels were significantly elevated at 8 h post-infection, with mRNA peaking at 12 h. While a temporal lag between transcription and translation is typically expected, we propose that several factors specific to our experimental model contribute to this observed timeline. First, previous studies using immortalized A549 cells reported a rapid *TLR4* mRNA peak as early as 2 h (Regueiro et al., 2009). However, our model utilizes primary HBE cells, which maintain more complex innate immune regulatory networks and higher activation thresholds than adenocarcinoma cell lines (Maris et al., 2006). Second, the thick CPS of our wild-type strain (ATCC 43816) likely acts as a physical shield, masking membrane-bound LPS and delaying initial TLR4 recognition (Regueiro et al., 2006). This masking effect is supported by our finding that the acapsular mutant induced earlier and stronger responses. Therefore, the 12 h mRNA peak in our model likely represents a secondary, amplified wave of transcription. This establishes a sophisticated framework for the induction of hyper-inflammation that the pathogen first induces the production of the alarmin S100A8/A9 while simultaneously increasing the abundance of its receptor, TLR4. The subsequent autocrine signaling of S100A8/A9 on these TLR4-upregulated cells results in a greatly magnified inflammatory output. This integrated process of priming and triggering provides a compelling molecular explanation for the rapid and often excessive inflammation characteristic of severe *K. pneumoniae* pneumonia (Achouiti et al., 2012; Revenstorff et al., 2022).

The function of S100A8/A9 in bacterial pneumonia has been a subject of complex and sometimes conflicting reports (Kumar et al., 2023; Xie et al., 2023). A key study by Revenstorff et al. (2022) using a murine model of *K. pneumoniae* pneumonia demonstrated a protective role for S100A8/A9, where its absence led to increased bacterial dissemination and mortality. The findings in that study attributed the protective effect to the antimicrobial properties of S100A8/A9, such as zinc chelation, and its role in enhancing macrophage phagocytosis. The present study does not contradict these findings but rather uncovers a distinct, localized, and potentially pathogenic role for S100A8/A9. This apparent discrepancy likely arises from differences in the experimental context. The study by Revenstorff et al. (2022) focused on the systemic effects of myeloid-derived S100A8/A9 in an *in vivo* mouse model, where its antimicrobial functions are critical for controlling bacterial burden in distant organs. In contrast, the current work focuses on the initial host-pathogen interaction at the human epithelial barrier, where S100A8/A9 functions not as an antimicrobial, but as a DAMP that drives local inflammation. This suggests a dual, context-dependent function for S100A8/A9, acting systemically in a protective antimicrobial capacity but also locally as a potentially detrimental pro-inflammatory mediator. Our data align with other studies showing that excessive S100A8/A9 at inflammatory sites can drive tissue pathology (Huang et al., 2025; Wang et al., 2025).

The discovery of this epithelium-intrinsic inflammatory loop has profound implications for understanding the pathogenesis of *K. pneumoniae*. The bacterium is well-known for its polysaccharide capsule, a major virulence factor that provides an immune-evasive shield by masking underlying PAMPs like LPS (Regueiro et al., 2006; Zhou et al., 2025). Our results, showing that an acapsular mutant induces more S100A8/A9 than the wild-type strain, are consistent with this masking function (Schembri et al., 2005; Wu et al., 2009). This suggests that the encapsulated bacterium may initially dampen direct inflammatory responses to facilitate colonization, while simultaneously triggering a more insidious, host-driven inflammatory amplification circuit via the S100A8/A9-TLR4 axis. This delayed but powerful inflammatory burst, driven by the host own epithelial cells, could contribute to the extensive lung damage and acute respiratory failure seen in severe clinical cases (Dai and Hu, 2022; Holmes et al., 2023). The functional relevance of this pathway was confirmed by the demonstration that the S100A8/A9 loop is a major driver of neutrophil chemotaxis, a hallmark of bacterial pneumonia.

A notable limitation of our investigation is that the conclusions are derived from experiments in primary HBE cell cultures. The findings are based on an *in vitro* model using primary HBE cells. While this model is highly relevant for studying the initial epithelial response, it does not capture the complex interplay with other resident and recruited immune cells, such as alveolar macrophages and neutrophils, which are also major sources or responders to S100A8/A9 (Wang et al., 2018; Guo et al., 2021; Ji et al., 2024). Future studies using co-culture systems or *in vivo* models with cell-type-specific deletions of S100A9 or TLR4 would be necessary to validate these findings in a more complex tissue environment. Furthermore, this study utilized a single laboratory strain of *K. pneumoniae*. Given the vast diversity of clinical isolates, including hypervirulent and multidrug-resistant strains, future work should examine the conservation of this autocrine loop is a conserved mechanism across different pathotypes.

**Figure 7:**
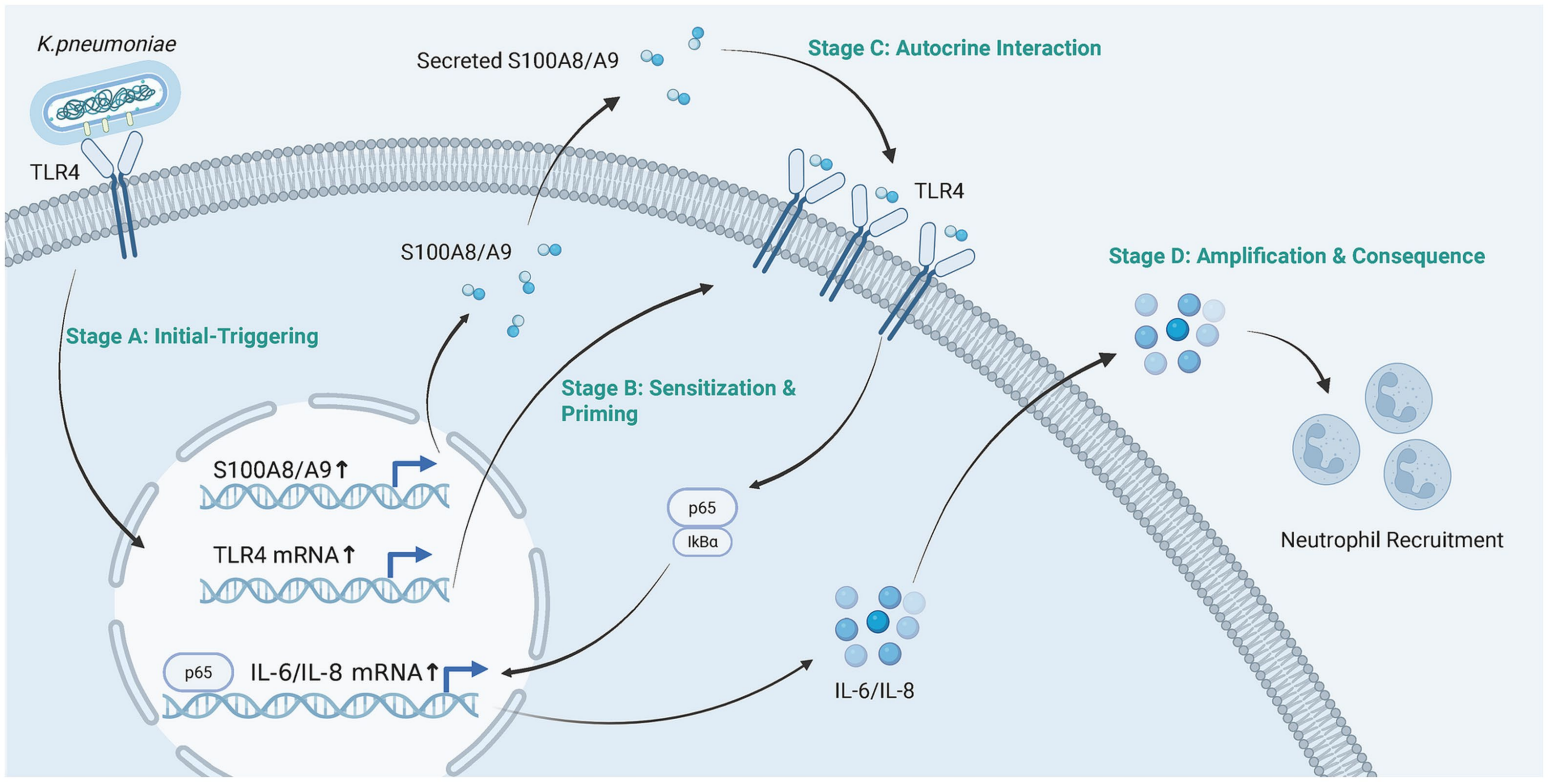
Proposed model of the S100A8/A9-mediated autocrine amplification loop in human airway epithelial cells during *K. pneumoniae* infection. Initial recognition of encapsulated *K. pneumoniae* by the airway epithelium triggers a primary transcriptional response, leading to the synthesis and secretion of the alarmin S100A8/A9 and a simultaneous upregulation of its cognate receptor, TLR4. This dual mechanism creates a primed state within the epithelium. The endogenously produced S100A8/A9 then acts back on the enriched TLR4 receptors in an autocrine or paracrine manner, activating the canonical NF-κB signaling pathway (characterized by IκB degradation and p65 nuclear translocation). This positive feedback loop significantly magnifies the production of pro-inflammatory cytokines such as IL-6 and IL-8, ultimately orchestrating massive neutrophil recruitment and driving the hyper-inflammation observed in severe pneumonia. Created in BioRender [You (2026) https://BioRender.com/qo9szis].

In conclusion, this study repositions the airway epithelium from a passive barrier to an active conductor of the early inflammatory orchestra during *K. pneumoniae* infection (Figure 7). The identification of an S100A8/A9-driven autocrine amplification loop provides a new framework for understanding the immunopathology of severe bacterial pneumonia. This epithelium-intrinsic pathway represents a promising target for novel host-directed therapies aimed at mitigating excessive lung inflammation and injury, a critical need in an era of increasing antimicrobial resistance.

## Data Availability

The raw data supporting the conclusions of this article will be made available by the authors, without undue reservation.
