# Supplementary material for: Klebsiella pneumoniae infection induces an S100A8/A9-mediated autocrine loop in human airway epithelium to amplify inflammation
Source: Front Microbiol. 2026 Mar 3;17:1768140. doi: 10.3389/fmicb.2026.1768140 (PMC12992257; doi:10.3389/fmicb.2026.1768140)
Supplement: Supplementary file 1 [file Data_Sheet_1.docx]

**Zheng et al. Supplemental Materials**

**Methods S1. Gentamicin Protection Assay**

To quantify bacterial internalization, HBE cells were infected with WT *K. pneumoniae* at an MOI of 100 for 1 h at 37°C. Following infection, cells were washed three times with PBS to remove non-adherent bacteria and then incubated with BEGM basal medium containing gentamicin (100μg/mL) for 1 h to eliminate extracellular bacteria. After three additional washes, cells were lysed with 0.1% Triton X-100 for 15 min. The cell lysates were serially diluted and plated on LB agar to determine the number of colony-forming units (CFU). Internalization efficiency was calculated as the percentage of the initial inoculum recovered from the intracellular compartment (Recovery rate % = [recovered CFU / initial inoculum CFU] ×100%.

**Supplementary Methods S2. TNF-α Stimulation Assay**

As a specificity control for general immunological signaling, HBE cells transfected with siCtrl or siS100A9 were stimulated with recombinant human TNF-α (10 ng/mL, R&D Systems) for 24 h. Cell culture supernatants were harvested, and the concentrations of IL-8 and IL-6 were determined by ELISA as described in the main text.


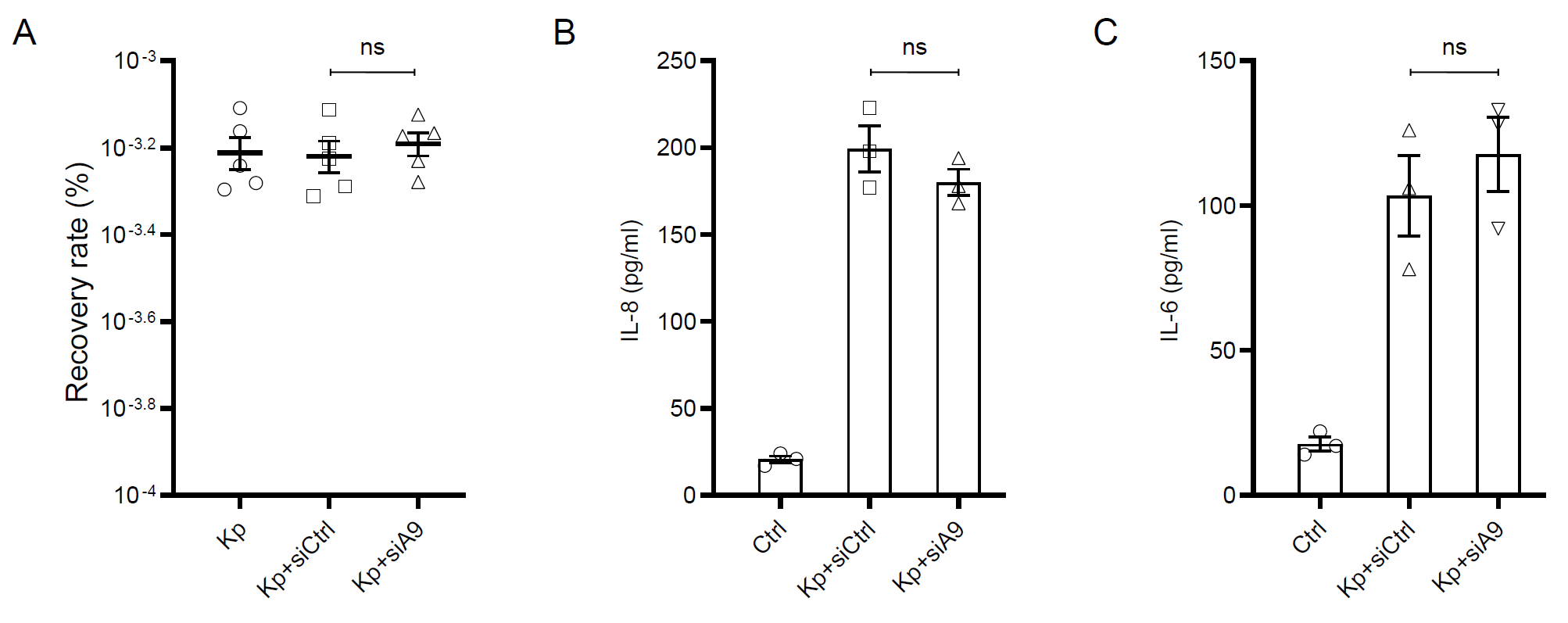


**Figure S1. S100A9 knockdown does not alter bacterial internalization or general cytokine responsiveness in HBE cells.** (A) Internalization efficiency of WT *K. pneumoniae* in HBE cells transfected with siCtrl or siS100A9, determined by gentamicin protection assay at 1 h post-infection. Data are expressed as recovery rate (% of inoculum). (B-C) Secretion of (B) IL-8 and (C) IL-6 by siCtrl- or siS100A9-transfected HBE cells following stimulation with TNF-α (10ng/mL) for 24 h. Individual data points represent independent biological replicates (n=3 or n=5). N.S., not significant (P>0.05).
